# Efficient azobenzene co-sensitizer for wide spectral absorption of dye-sensitized solar cells†

**DOI:** 10.1039/c7ra12229b

**Published:** 2018-02-07

**Authors:** L. Y. Zhang, S. J. Zou, X. H. Sun

**Affiliations:** School of Chemical Engineering, Northeast Electric Power University Jilin 132012 P. R. China zhanglingyun@vip.sina.com +86-432-64806371 +86-432-64807273

## Abstract

Two azobenzene dyes, [Cu(azobenzene-4,4′-dicarboxylate) diethylenediamine]_*n*_ (ADD), [Cd(4,4′-diazenediyldibenzoato)(H_2_O)]_*n*_ (DDB), have been designed, synthesized, and characterized as efficient co-sensitizers for dye-sensitized solar cells (DSSC). The optical, charge-transfer, electrochemical and photovoltaic properties of ADD and DDB are investigated by UV-visible spectroscopy, transient surface photovoltage measurement, cyclic voltammetry, and photocurrent–photovoltage measurement. The combination of ADD and DDB in DSSC leads to a wide spectral absorption over the whole visible range (350–700 nm). DSSC with ADD and DDB exhibits a short-circuit photocurrent density as high as 16.96 mA cm^−2^, open-circuit photovoltage of 0.73 V, a fill factor of 0.57, and overall light conversion efficiency of 7.1% under standard global AM1.5 solar irradiation conditions.

## Abstract 2

The combination of two azobenzene dyes leads to a wide spectral absorption and overall light conversion efficiency of 7.1%.

## Graphical abstract

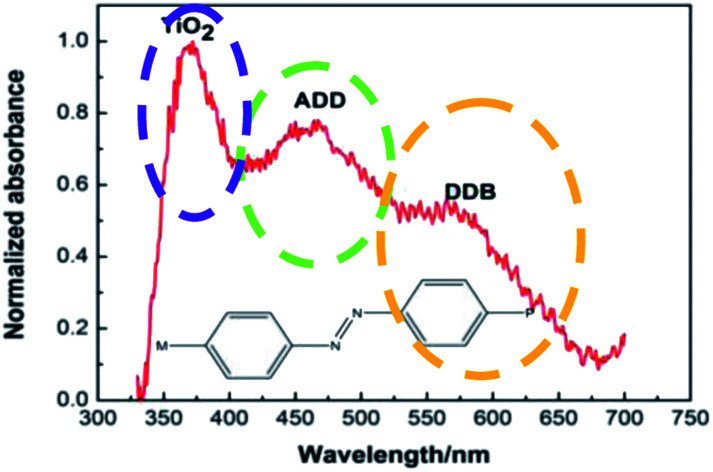

## Introduction

In the face of limited fossil fuels and the disastrous environmental problem, people realize the urgent need to develop renewable energy resources for the increasing global energy demand. Solar energy is the most hopeful for supplying sustainable energy because it is a type of clean and renewable energy. Dye-sensitized solar cells (DSSC) have attracted much attention as a promising technology for the performance/price ratio cells.^1^ The dye is a key component which determines the efficiency of DSSC as it absorbs sunlight, excites electrons and injects electrons into the conductor band of the semiconductor. In the future, cost-effective organic dyes will play a pivotal role in the large-scale production and application of DSSC. The development of new dyes is all through the focus of DSSC.

The ideal dye can absorb all the light in solar spectrum.^2–5^ So far, the most close to the ideal dye are distyryl-substituted boradiazaindacene dye (BODIPY) and “black dye”.^6,7^ BODIPY has panchromatic spectral absorption between 400 and 800 nm in visible light region. The black dye achieves very efficient sensitization over the whole visible range extending into the near-IR region up to 920 nm, yielding an overall conversion efficiency of 10.4%. Another way to get the full absorption spectrum is to use a co-sensitization of multi-dyes with different absorption ranges.

Azobenzene complex is made up of benzene ring linked by a nitrogen double bond. Azobenzene complex occurs n–π* and π–π* transition by irradiation. The absorption bands of the n–π* and π–π* transition are located in the ultraviolet and visible regions, respectively. By introducing different light absorbing groups in the benzene ring, the absorption range and intensity of azobenzene complex can be controlled. Azobenzene complex with above optical property may be a potential sensitizer for DSSC.

In this paper, two novel azobenzene dyes (ADD and DDB) were designed, synthesized, and co-sensitized TiO_2_ photoanode. The properties of ADD and DDB were investigated. The combine of ADD and DDB achieved very efficient sensitization over the whole visible range (350–700 nm).

## Experimental

All the starting materials were reagent grade and used as purchased without further purification. Distilled water was used throughout. The radical ligand H_2_L, azobenzene-4,4′-dicarboxylate, was prepared according to the literature.^8^

### Synthesis of ADD

A mixture containing H_2_L (5 mg, 0.019 mmol), CuCl_2_·6H_2_O (60 mg, 0.36 mmol), *N*,*N*′-dimethylacetamide (DMA 15 mL), H_2_O (2 mL) and ethylenediamine (en 0.01 mL) was stirred at room temperature for 1 h until a clear red solution formed. It was then transferred into a Parr Teflon-lined stainless steel vessel (25 mL) and heated to 120 °C for 72 h under autogenous pressure. Afterwards, it was cooled to 25 °C at a rate of 4 °C h^−1^. Brown flake crystals were obtained after washing with DMA and drying in air, in about 42% yield (based on Cu). Anal. calcd for C_16_H_16_N_4_CuO_4_ (391.87): C 49.04; H 4.12; N 14.30%; found: 48.97; H 4.09; N 13.68%. IR data (KBr pellet, cm^−1^, Fig. S1†): 3316 (s), 1612 (s), 1607 (s), 1554 (s), 1515 (w), 1368 (s), 1293 (w), 1214 (w), 1153 (w), 1094 (w), 1049 (s), 1008 (m), 870 (m), 783 (s), 703 (s).

### Synthesis of DDB

**Scheme 1:**
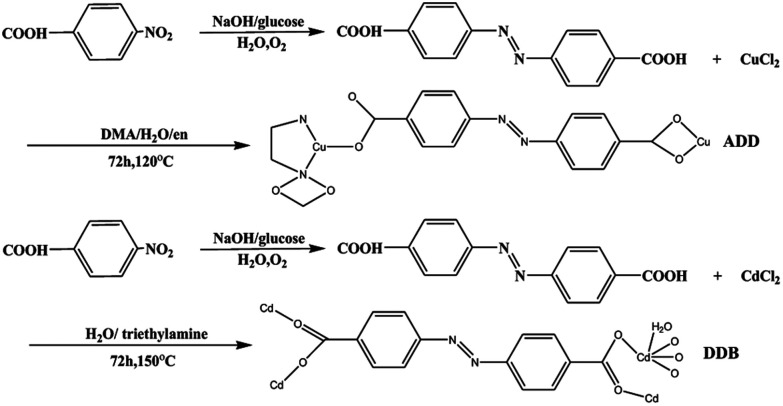
Synthetic routes to ADD and DDB.

The synthesis of DDB is slightly different from the literature.^9^ A mixture containing H_2_L (5 mg, 0.019 mmol), CdCl_2_·2.5H_2_O (30 mg, 0.15 mmol), H_2_O (12 mL) and triethylamine (0.05 mL) was stirred at room temperature for 1 h until a clear solution formed. It was then transferred into a Parr Teflon-lined stainless steel vessel (25 mL) and heated to 150 °C for 72 h under autogenous pressure. Afterwards, it was cooled to 25 °C at a rate of 4 °C h^−1^. Brown flake crystals were obtained after washing with DMA and drying in air. The synthetic routes of ADD and DDB are depicted in Scheme 1.

### Fabrication of DSSC

A colloidal solution of TiO_2_ was obtained by being coated on the fluorine-doped tin oxide (FTO) conducting glass by screen printing and then dried for 6 min at 125 °C. The TiO_2_ electrodes were gradually heated under an air flow at 325 °C for 5 min, at 375 °C for 5 min, at 450 °C for 15 min, and at 500 °C for 15 min. The area of film was 0.25 cm^2^. The film electrodes were soaked in the DDB solution (concentration: 5 × 10^−4^ M, solvent: absolute ethanol) for 24 h and the ADD solution (concentration: 5 × 10^−4^ M, solvent: absolute ethanol) for 24 h at room temperature, respectively. Then, the co-sensitized films were dried in air. The counter electrodes were thermally platinized conducting glass (5 mM H_2_PtCl_6_ in dry isopropanol, heated at 400 °C for 10 min). The electrolyte was consisted of 0.5 M LiI, 0.05 M I_2_, 0.1 M 4-*tert*-butylpyridine in 1 : 1 (volume ratio) acetonitrile–propylene carbonate.

## Characterization

### X-ray structure determination

A suitable crystal with dimensions of 0.24 mm × 0.12 mm × 0.05 mm was selected for single-crystal X-ray diffraction and the data were collected at 296(2) K on Bruker Apex II CCD area-detector diffractometer (Mo Kα, 0.071073 nm). A total of 10 123 reflections were collected by a *φ*–*ω* scan mode at room temperature in the range of 1.65< *θ* < 28.46° including 4016 independent ones with *R*_int_ = 0.0199. Data reductions and absorption corrections were performed using SADBAS program. The structure was solved by direct methods and refined using full-matrix least-squares methods on *F*^2^ with the SHELXS-97 program. All non-hydrogen atoms were refined anisotropically. All hydrogen atoms were located geometrically by the program olex 2. The CCDC number of ADD is 881781. The detailed crystallographic data and structure refinement parameters for ADD are summarized in Table S1.† Selected bond lengths and bond angles of ADD are listed in Table S2.† Hydrogen bond distances and angles for ADD are listed in Table S3.†

### Photoelectrochemical measurements

UV-visible absorption spectra of the samples were measured at room temperature on U-3010 spectrophotometer (Hitachi, Japan) with an integrating sphere (model 130-0632). Cyclic voltammetry experiment was performed with a three-electrode system, a platinum working electrode, a platinum counter electrode, and an Ag/AgCl reference electrode. The potentials were referenced to the ferrocene/ferrocenium (Fc^+^/Fc) couple.

The photocurrent–photovoltage (*J*–*V*) curves of the sealed cells were measured under AM 1.5 illumination (100 mW cm^−2^) by using a solar simulator. Electrochemical impedance measurements were carried out by electrochemical work-station (chi660d, Chenhua, China) with three-electrode system in the dark. Frequency range was 0.05–105 Hz, and the applied potential was generally between 0–0.700 V. The IPCE spectra were measured with a Model SR830 DSP Lock-In Amplifier and a Model SR540 Optical Chopper (Stanford Research Corporation, USA), a 7IL/PX150 renon lamp as light source, and a 7ISW301 Spectrometer.

## Results and discussion

### Crystal structure

**Fig. 1:**
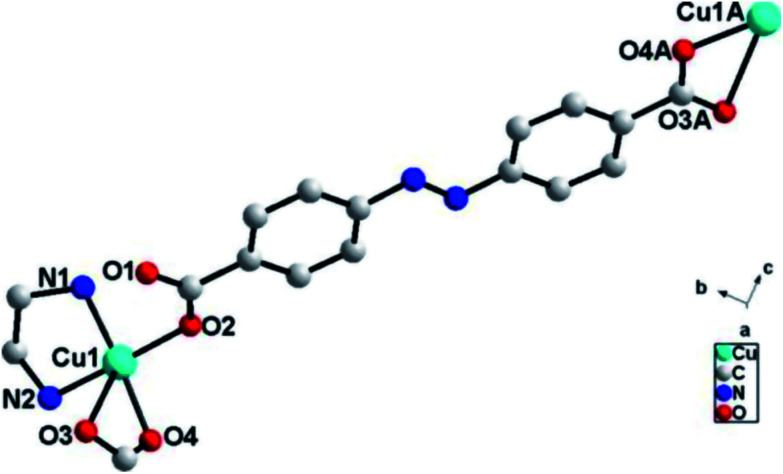
The coordination environments of Cu1 in ADD (symmetry code: A, −0.5 − *x*, −*y*, 0.5 + *z*).

Single crystal X-ray diffraction analysis reveals that ADD crystallizes in the orthorhombic space group *P*2_1_2_1_2_1_. The asymmetric unit of ADD contains one Cu(ii) atom, one L^2−^ ligand and one en molecule. ADD is coordinated into a distorted rectangular pyramid geometry by two nitrogen atoms (N(1) and N(2)) from one en, one oxygen atom (O(2)) from carboxylic acid group of one L^2−^ ligand and two oxygen atoms (O(3) and O(4)) from carboxylic acid group of another L^2−^ ligand (Fig. 1). The distances of Cu–N/O [0.193(13)–0.270(16) nm] and the angle of O/N–Cu–O/N [53.61(5)–92.55(6)°] are in the normal range of those observed in reported Cu(ii) compounds.^10^

**Fig. 2:**
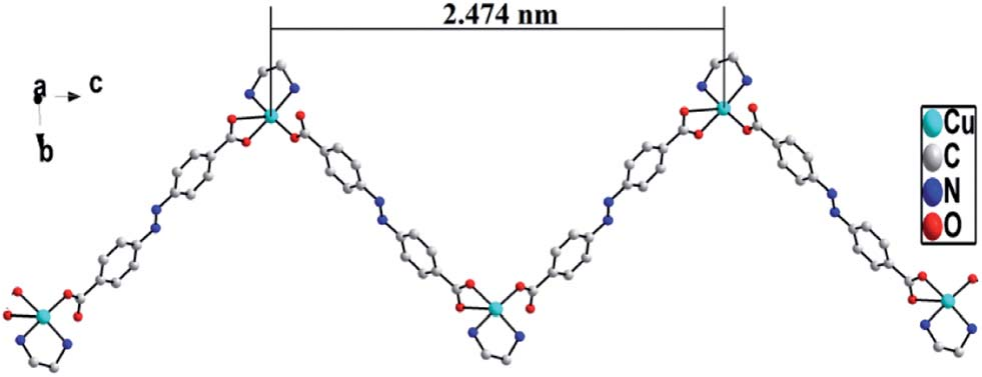
1D chain structure in ADD.

In ADD, L^2−^ ligand exhibits a μ2-terdentate bridging coordination mode, in which the carboxylic acid oxygen atom (O(2)) coordinate one Cu(ii) cations, and the oxygen atoms (O(3)A and O(4)A) of another carboxylic acid group adopt chelating coordination model to link another Cu(ii) cations. As shown in Fig. 2, Cu(ii) cations are linked into an infinite 1D zig-zag chain along *c* axis by the carboxylic oxygen groups of L^2−^ ligands with the adjacent sinusoidal ruffling motif Cu⋯Cu distance of *ca.* 2.474 nm. While en molecules chelate to Cu(ii) cations from both sides of the chain.

**Fig. 3:**
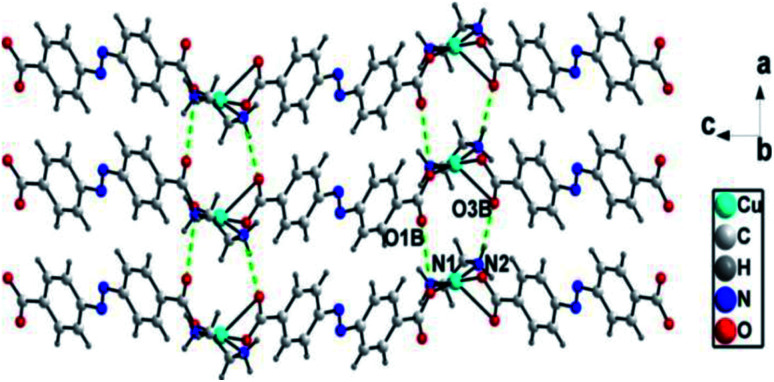
1D chains are connected into 2D supramolecular layer structures through hydrogen bonding interactions in ADD (symmetry code: B, 1 + *x*, *y*, *z*).

There exist N–H⋯O [N(1)⋯O(1)B = 0.294(3) nm, N(1)–H(1B)⋯O(1)B = 135°; N(2)⋯O(3)B = 0.289(2) nm, N(2)–H(2A)⋯O(3)B = 168°] hydrogen bonds between adjacent zig-zag chains, linking the 1D chains into a two-dimensional (2D) layer structure (Fig. 3).

We examined the structural homogeneity of bulk powder sample of ADD through comparison of experimental and simulated PXRD patterns. The peak positions of the experimental patterns are nearly in agreement with those of the simulated one generated from single-crystal X-ray diffraction data (Fig. S2†), suggesting that the product of ADD is pure single phase.

To estimate the stability of ADD and DDB, the thermal behavior was carried out by TGA under N_2_ atmosphere in the temperature range from 20 to 800 °C at a rate of 10 °C min^−1^. For ADD, the weight loss of 80.56% from 295 to 382 °C corresponds to the loss of the ligand and en for ADD (calculated value is 79.67%) (Fig. S3†). For DDB, the first mass loss of 5.32% occurs between 150 to 226 °C, corresponding to the release of its one coordinated water molecule (calcd 4.63%) (Fig. S4†). The solid then continues to lose mass from 387 to 725 °C, corresponding to the decomposition of one L^2−^ ligand (obsd 62.34%, calcd 62.23%). Finally, a plateau occurs from 740 to 800 °C. The residue equals 32.34%, which is attributed to CdO (calcd 32.21%).

**Fig. 4:**
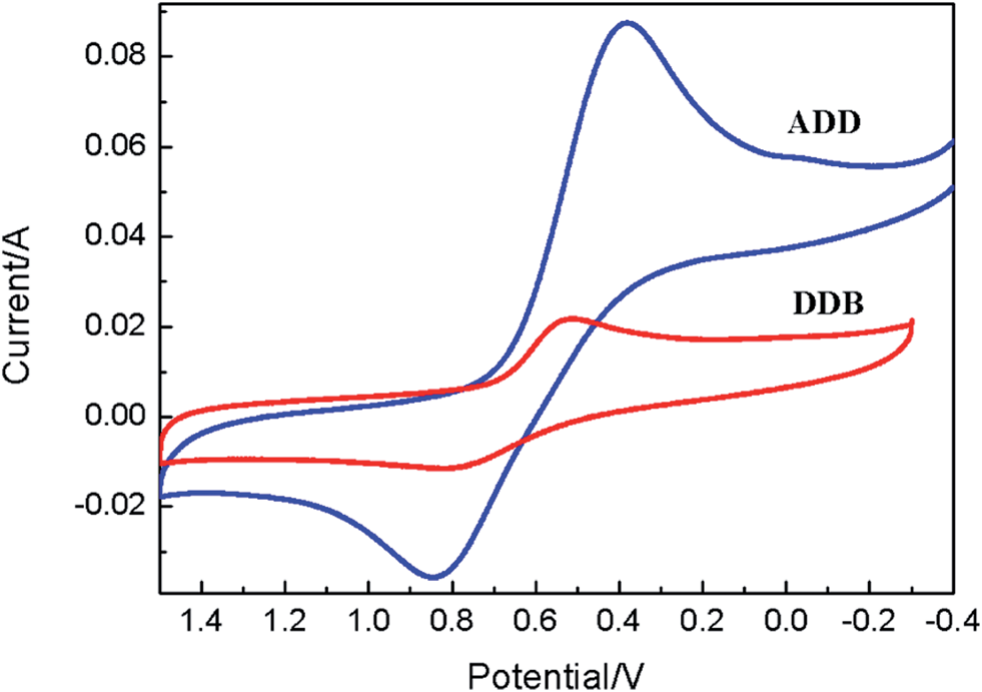
Cyclic voltammograms of the ADD and DDB in CH_2_Cl_2_ containing 0.1 M TBAPF_6_ solution.

Cyclic voltammetry is used to study the electrochemical properties of ADD and DDB, the mechanism of electron injection from the ADD and DDB to conductor band of TiO_2_, the oxidation and reduction regeneration of ADD and DDB in the electrolyte. Fig. 4 shows the cyclic voltammetry of ADD and DDB in CH_2_Cl_2_ solution. ADD and DDB exhibit a reversible oxidation–reduction behavior. The ground-state oxidation potential (*E*_ox_) corresponding to the high occupied molecular orbital (HOMO) level is determined by the peak potential of CV.^11^*E*_ox_ of ADD and DDB are 0.84 and 0.79 V (*vs.* NHE) respectively, which are more positive than the redox potential of I_3_^−^/I^−^ couple (0.4 V *vs.* NHE).^12^ It suggests that the oxidized ADD and DDB formed after electron injection into the conductor band of TiO_2_ should be thermodynamically reduced by I_3_^−^/I^−^ in the electrolyte. The absorption threshold of ADD is 550 nm, which corresponds to the band gap energy of 2.25 eV according to eqn (1).^13^

1 *E*_g_ (eV) = 1240/*λ* (nm)

The excited-state redox potential (*E*_red_) of ADD corresponding to the low unoccupied molecular orbital (LUMO) level is calculated to be −1.41 V according to eqn (2).^14^

2 *E*_red_ = *E*_ox_ + *E*_g_

**Fig. 5:**
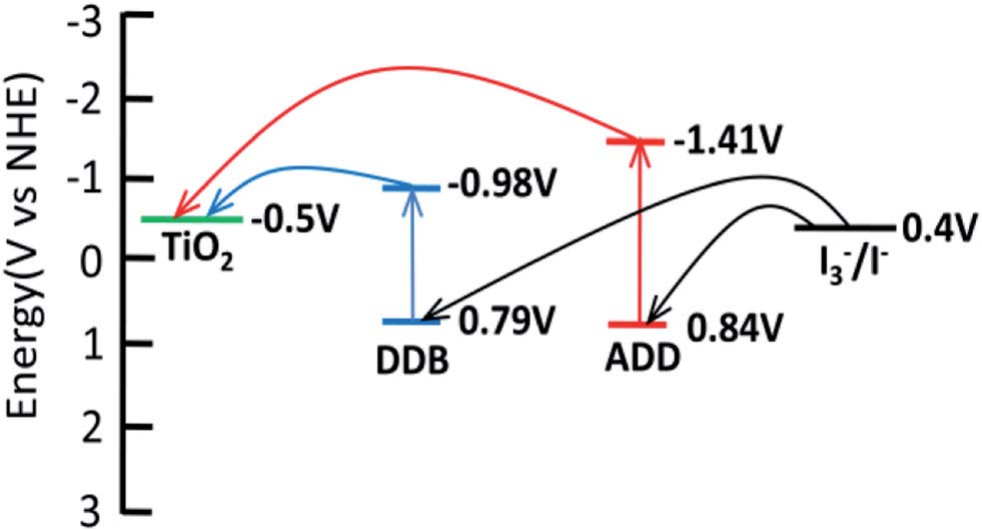
LUMO and HOMO energy levels of ADD and DDB with respect to the conductor band (CB) edge and the valence band (VB) edge energies of TiO_2_ and the I_3_^−^/I^−^ redox potential.

The absorption threshold of DDB is 700 nm. *E*_red_ of DDB is −0.98 V. *E*_red_ of ADD and DDB is more negative than the bottom of conductor band of TiO_2_ (−0.5 V *vs.* NHE), which confirms that the electron injection from excited state is thermodynamically favorable. Fig. 5 shows the schematic energy levels of ADD and DDB based on the absorption and the electrochemical data. The exciting, electron injecting and redox regenerating of ADD and DDB are exhibited in Fig. 5, indicating ADD and DDB can efficiently sensitize TiO_2_ photoanode of DSSC.

**Fig. 6:**
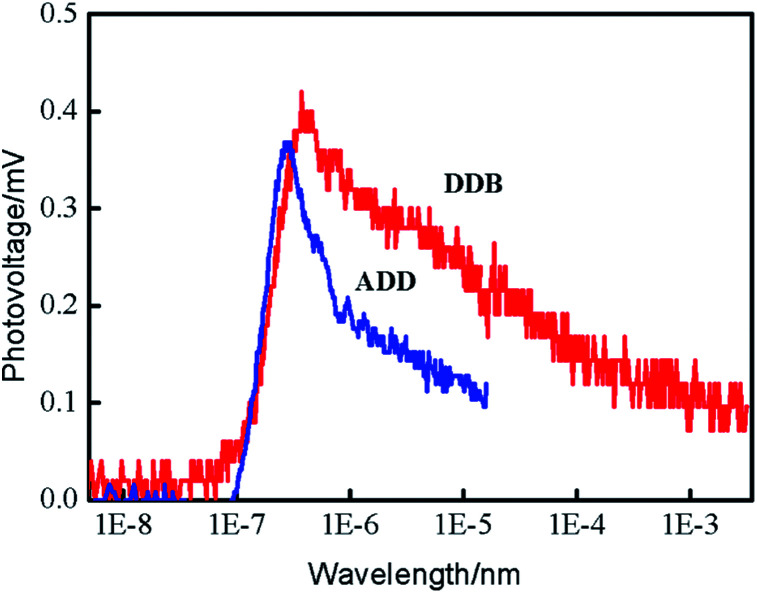
The transient surface photovoltage spectra of ADD and DDB.

Transient surface photovoltage (TPV) measurement was performed to study the mechanisms of charge separation, diffusion and recombination processes. Fig. 6 shows the positive TPV response for ADD and DDB. The layer thickness of materials is much greater than the diffusion distance of excess electrons. Therefore, the TPV shape depends not on the layer thickness of materials but the carrier changes with the time. The positive TPV response implies that negative charges transfer towards the bottom and positive charges accumulate at the surface area. The electrons and holes diffuse from surface to bottom due to the concentration gradient. The diffusion coefficient of electrons is larger than that of holes. The TPV response of DDB is stronger than that of ADD. The enhanced TPV of DDB indicates that the separation of photo-generated electrons and holes is much more pronounced than that of ADD.^15^ Charge separation increased progressively from the instant at 10^−7^ s until it attained a saturation value. The TPV apexes on the time scale for ADD is smaller than that of DDB. This indicates that separation and diffusion of electrons and holes in ADD is faster than that in DDB. The recombination of electrons and holes is also faster than that in DDB. The lifetime of electrons in ADD is shorter than that in DDB.

**Fig. 7:**
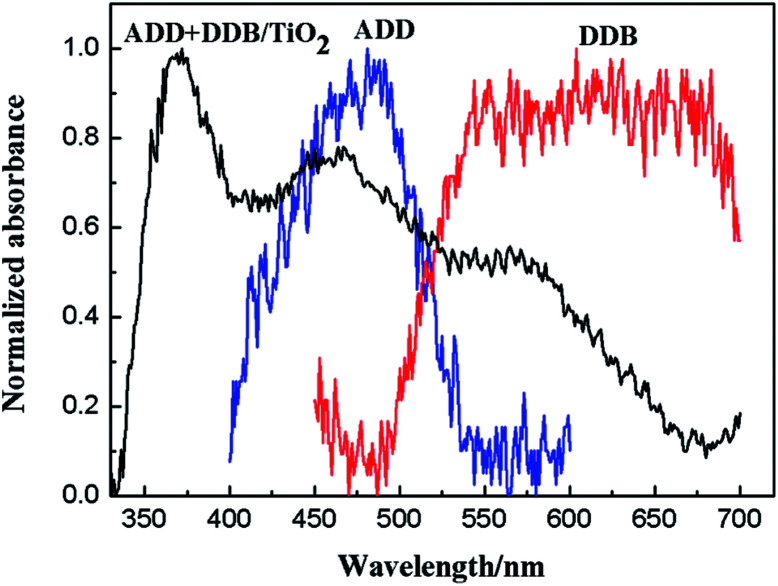
The UV-visible absorption spectra of ADD and DDB.

**Fig. 8:**
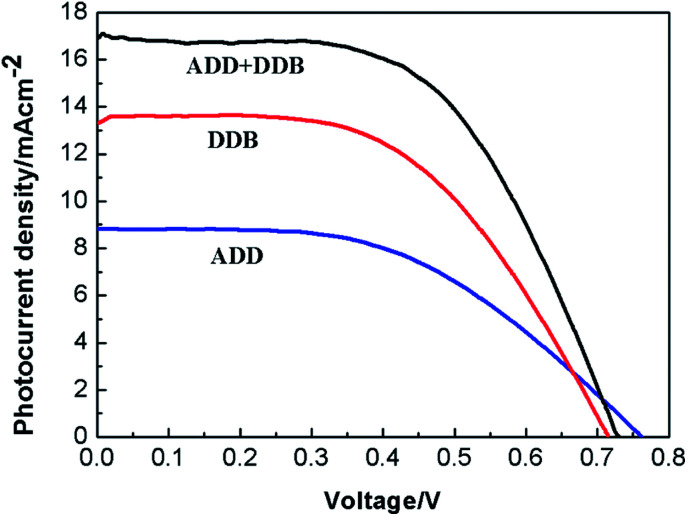
The current density *versus* voltage curves of DSSC based on ADD and DDB under 100 mW cm^−2^ illumination.

Fig. 7 shows normalized absorption spectra of ADD and of DDB in absolute ethanol solution (10^−5^ mol L^−1^). ADD shows absorbing response in the blue light region (400–550 nm) with maximum absorption peak at 480 nm. DDB shows wide and intense visible light absorption between 500 and 700 nm. The absorption bands of ADD and DDB are attributed to the π–π* transitions.^16,17^ Obviously, the combination of ADD and DDB can effectively broaden the absorption response of DSSC. As shown in Fig. 8, the absorption spectrum of ADD and DDB absorbed on the TiO_2_ film exhibits absorption response in 350–700 nm. The new absorption peak central at 370 nm is attributed to the absorption of TiO_2_. It is known that the dyes have the strong attractive forces among the molecules at the solid–liquid interface. The attractive forces can lead the shift of absorption peak. The attractive forces have two forms: red-shift J-aggregation and blue-shift H-aggregation. The maximum absorption peak of ADD attached to TiO_2_ film has obviously blue-shifted by 20 nm from 480 to 460 nm compared to that in absolute ethanol solution, owing to dye anchoring and H-aggregation on TiO_2_ semiconductor surface. As a whole, the combination of ADD and DDB broadens and intensifies the absorption of DSSC, which should be profitable for light harvesting and photocurrent generation in DSSC.

Fig. 8 shows the current density *versus* voltage curves of DSSC based on the films of ADD/TiO_2_, DDB/TiO_2_ and ADD + DDB/TiO_2_ under 100 mW cm^−2^ illumination. The photovoltaic performances of ADD/TiO_2_-based DSSC, *e.g.* the short photocurrent density (*J*_sc_), the open circuit voltage (*V*_oc_), the fill factor (FF), and overall energy conversion efficiency (*η*) are 8.83 mA cm^−2^, 0.77 V, 0.50, 3.4%, respectively. *J*_sc_, *V*_oc_, FF, and *η* of DDB/TiO_2_-based DSSC are 13.33 mA cm^−2^, 0.71 V, 0.55, 5.3%, respectively. *J*_sc_ of DDB/TiO_2_-based DSSC is bigger than that of ADD/TiO_2_-based DSSC. This is attributed to broaden absorption range of DDB in visible light region. *V*_oc_ is the difference potential between the electrochemical potential of the redox couple in the electrolyte and the conduction band edge of TiO_2_ photoanode.^18^ LUMO level of ADD is higher than that of DDB. The upward shift of the conduction band edge of TiO_2_ caused by ADD is much intense than that of DDB, so *V*_oc_ of ADD is higher than that of DDB. The photovoltaic performances of ADD + DDB/TiO_2_-based DSSC are obviously increased compared with that of single ADD or DDB. *J*_sc_ of ADD + DDB/TiO_2_-based DSSC (16.96 mA cm^−2^) has a remarkably increase compared relative to that of single ADD or DDB. This increase of *J*_sc_ indicates that ADD and DDB effectively co-sensitized TiO_2_ photoanode and resulted in a higher light harvesting. ADD and DDB complement each other in solar spectrum. This co-sensitization of ADD and DDB is an efficient method of improving efficiency. The overall solar conversion efficiency of DSSC based on ADD + DDB/TiO_2_ tremendously increases to 7.1%.

**Fig. 9:**
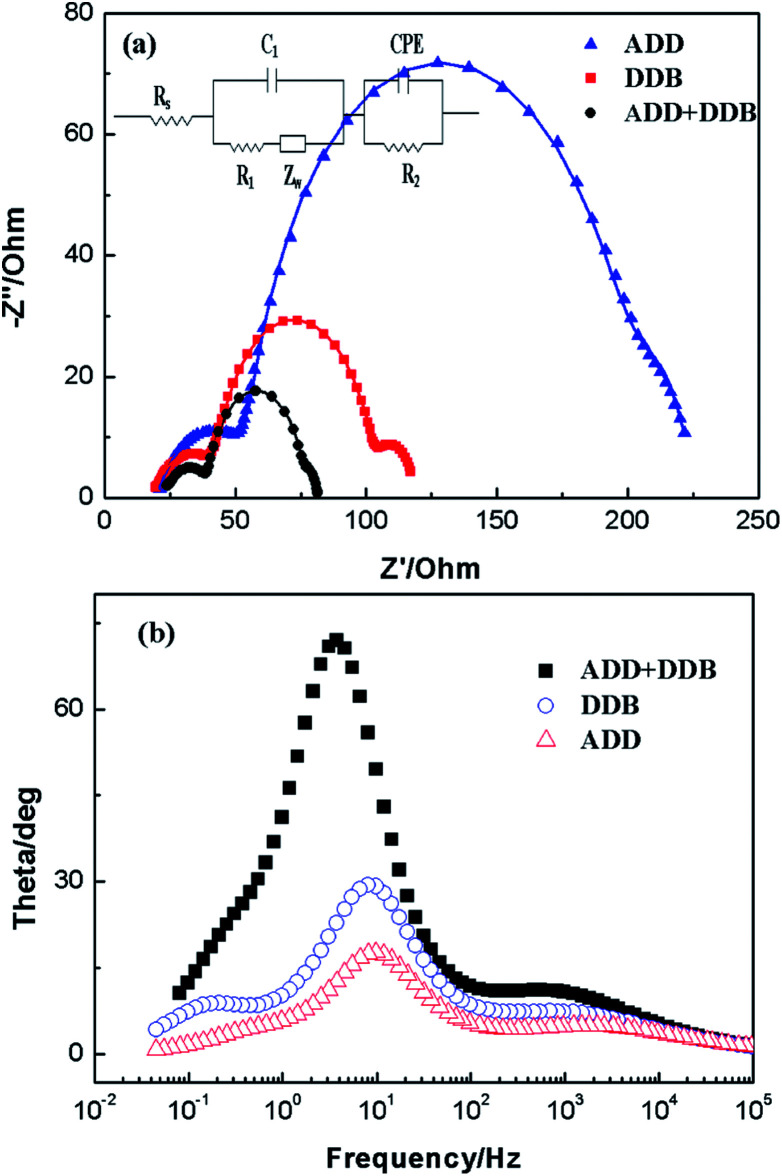
Impedance spectra of DSSC based on AAD, DDB and ADD + DDB in dark. (a) Nyquist plots; (b) Bode phase plots.

Electrochemical impedance spectroscopy (EIS) has been widely used to correlate device structure with a suitable model for the study of kinetics of electrochemical and photoelectrochemical processes occurring in DSSC. Fig. 9 shows electrochemical impedance spectroscopy of ADD, DDB and ADD + DDB based on DSSC in the dark. In Fig. 9, experimental data are represented by symbols while the solid lines correspond to the fit obtained by Zsimpwin software using the equivalent circuit *R*_s_(*C*_1_(*R*_1_*O*_1_))(*R*_2_*Q*_2_).^19,20^ The spectra show two semicircles at medium frequency region and high frequency region. The equivalent circuit is a useful tool for understanding the output performance of solar cells and for further comprehension of the electrical behavior of DSSC. In equivalent circuit, the symbols *R* and *C* describe resistance and capacitance, respectively; *O*, which depends on the parameters *Y*_o,1_ and *B*, accounts for a finite-length Warburg diffusion (*Z*_Dif_), and *Q* is the symbol for the constant phase element, CPE (its parameters are *Y*_o,2_ and *n*).^19^*Y*_o,1_ and *Y*_o,2_ are Warburg coefficients at the photoanode/dye/electrolyte interface and the Pt/electrolyte interface, respectively, corresponding to the module 1 and 2 in the equivalent circuit. *R*_Dif_ = *B*/*Y*_o,1_, *B* is parameter. Table 1 lists parameters obtained by fitting the impedance spectra of composite solar cells using the equivalent circuit. The series resistance of the system (*R*_s_), can account for the resistance of polymer electrolyte, the resistance within the photoelectrode film and the FTO electrode, as well as contacts. A semicircle observed at high frequency is associated to the capacitance and resistance at Pt/electrolyte interface (CPE and *R*_2_ elements).^19,21^ For the elements related with Pt/electrolyte interface, *R*_2_ of ADD, DDB, and ADD + DDB is 38.51 Ω, 26.72 Ω, and 18.92 Ω, respectively. The decrease of *R*_2_ means that much more I_3_^−^ at the Pt electrode surface to take place redox reaction and hence the FF of the DSSC. The large semicircle at the medium frequency is attributed to the electron transfer process at the dye/TiO_2_/electrolyte interface (capacitance, *C*_1_; charge transfer resistance, *R*_1_).^20,22^*C*_1_ of ADD, DDB, and ADD + DDB is 29.51 × 10^−5^, 32.97 × 10^−5^, and 47.91 × 10^−5^, respectively. The increasing film capacitance means more electrons at the interface of dye/TiO_2_/electrolyte as the light absorption is hence for DSSC. The decreasing value of *R*_1_ means that the resistance is low at the interface of dye/TiO_2_/electrolyte. This is favorable for electron transmission.

**Table 1:** Parameters obtained by fitting the impedance spectra of composite solar cells using the equivalent circuit

DSSC	*R*_s_ Ω^−1^	*C*_1_ F^−1^	*R*_1_ Ω^−1^	*Y*_o,1_ S^−1/2^	*B*/Ω S^1/2^	*R*_2_/Ω	*Y*_o,2_/S^1/2^	*n*
ADD	19.89	29.51 × 10^−5^	138.4	45.25 × 10^−3^	1.24	38.51	12.64 × 10^−5^	0.8
DDB	18.46	32.97 × 10^−5^	55.72	91.35 × 10^−3^	1.58	26.72	17.39 × 10^−5^	0.8
ADD + DDB	22.60	47.91 × 10^−5^	33.66	15.18 × 10^−2^	0.95	18.92	18.07 × 10^−5^	0.8

The high film capacitance and the low resistance are favorable for improving the performances of DSSC. Meanwhile, the recombination characteristic peak of DSSC based on ADD, DDB, and ADD + DDB is 9.8, 8.1, and 3.7 Hz. This indicates electron lifetime in ADD + DDB DSSC is longest. The high film capacitance and low resistance, low recombination characteristic frequency makes the highest *η* possible. Therefore, *η* of DSSC based ADD + DDB is highest.

## Conclusions

In summary, we have designed and synthesized two new azobenzene dyes, ADD and DDB as the sensitizer for DSSC application. The combination of ADD and DDB broadens the spectral response, prevents dye aggregation and suppresses charge recombination. DSSC based on ADD + DDB dyes yield overall solar conversion efficiency of 7.1%. This result suggests azobenzene dyes are potential high efficient organic sensitizers. More work should be processed in aspect of optimizing the structure of azobenzene dyes for high-performance DSSC in future.

## Conflicts of interest

There are no conflicts to declare.

## Supplementary Material

RA-008-C7RA12229B-s001
