# Supplementary material for: Efficient azobenzene co-sensitizer for wide spectral absorption of dye-sensitized solar cells
Source: RSC Adv. 2018 Feb 7;8(12):6212–7. doi: 10.1039/c7ra12229b (PMC9078221; doi:10.1039/c7ra12229b)
Supplement: RA-008-C7RA12229B-s001 [file RA-008-C7RA12229B-s001.pdf]

Electronic Supplementary Material (ESI) for RSC Advances.
This journal is © The Royal Society of Chemistry 2018

Table S1 Crystal data collections and structure refinements for ADD

compound 1			
Formula	C16H16N4CuO4	Crystal system	Orthorhombic
Fw	391.87	V /nm3	1.621(2)
Space group	P212121	Temperature (K)	296(2)
a/nm	0.533(4)	α/°	90.00
b/nm	1.228(10)	β/°	90.00
c/nm	2.474(19)	γ/°	90.00
Z	4	Dc/Mg m–3	1.606
F(000)	804	μ/mm–1	1.378
θ range (°)	1.65–28.46	Reflections collected	10123
Independent reflections (Rint)	4016(0.0199)	GOF	1.035
Final Ra,b indices [I>2σ(I)]	R1 = 0.0259<br>wR2 = 0.0639	R indices (all data)	R1 = 0.0321<br>wR2 = 0.0666

a R1= ∑||Fo| – |Fc||/∑|F|. b wR2 = |∑w(|Fo|2 – |Fc|2)2/∑|w(F)2|1/2

Table S2 Bond lengths (nm) and angles (°) of ADD

compound 1					
Bond	Dist.(nm)	Bond	Dist.(nm)	Bond	Dist.(nm)
Cu1-O2	0.193(13)	Cu1-N2	0.200(15)	Cu1-N1	0.198(14)
Cu1-O4	0.195(12)	N3-N4	0.123(3)		
Angle	(º)	Angle	(º)	Angle	(º)
O2-Cu1-O4	91.85(6)	O2-Cu1-N2	171.41(7)	N1-Cu1-N2	84.19(7)
O2-Cu1-N1	92.55(6)	O4-Cu1-N2	91.39(6)	O4-Cu1-N1	175.58(6)

Table S3 Hydrogen bond distances (nm) and angles (°) in ADD

D-H	d(D-H)	d(H···A)	∠DHA	d(D-A)	A
N(1)-H(1B)	0.090	0.223	135	0.294(3)	O(1)B
N(2)-H(2A)	0.090	0.200	168	0.289(2)	O(3)B
C(15)-H(15A)	0.097	0.253	139	0.332(2)	O(4)C

symmetry code: B, 1+x, y, z; C, 1–x, 0.5+y, 0.5–z

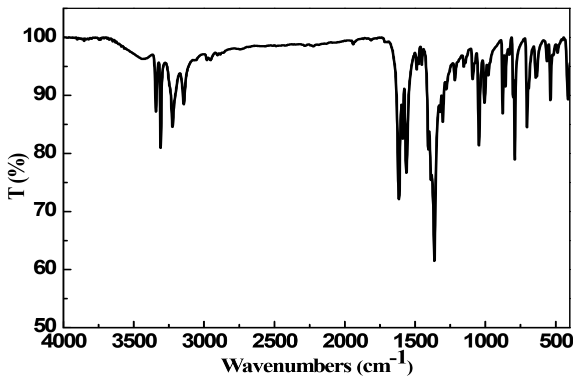

Fig.S1. IR curve of ADD

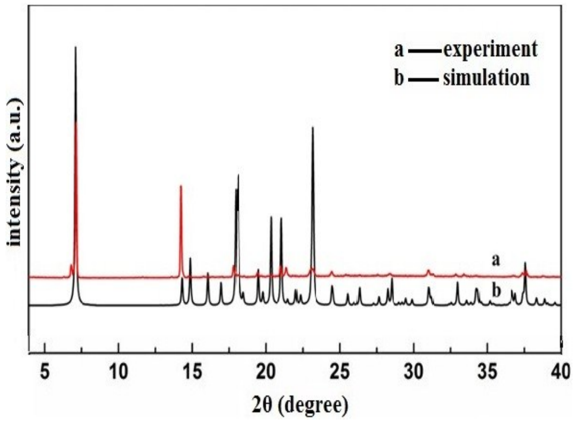

Fig.S2. The experimental (a) and simulative (b) powder X-ray diffraction patterns for ADD

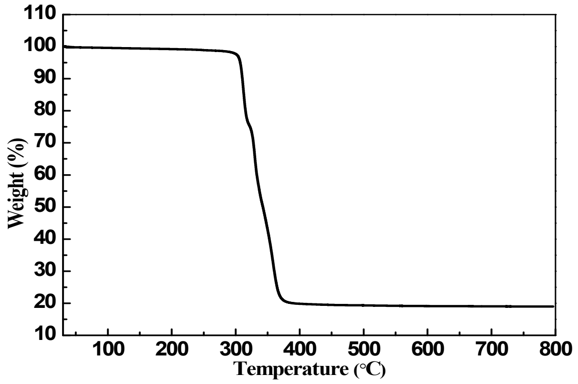

Fig.S3. TGA curve of ADD

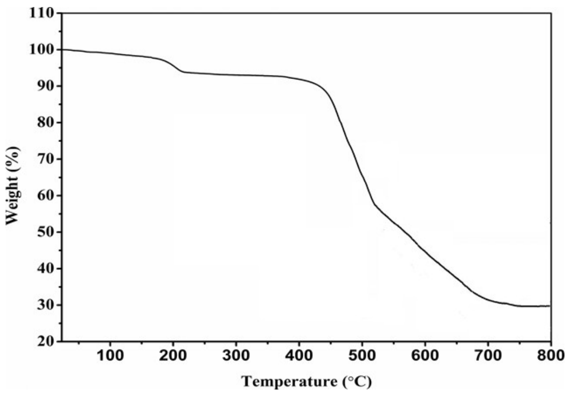

Fig.S4. TGA curve of DDB
